# Supplementary material for: Suppression of SRCAP chromatin remodelling complex and restriction of lymphoid lineage commitment by Pcid2
Source: Nat Commun. 2017 Nov 15;8:1518. doi: 10.1038/s41467-017-01788-7 (PMC5686073; doi:10.1038/s41467-017-01788-7)
Supplement: Supplementary file 1 — Supplementary Information [file 41467_2017_1788_MOESM1_ESM.pdf]

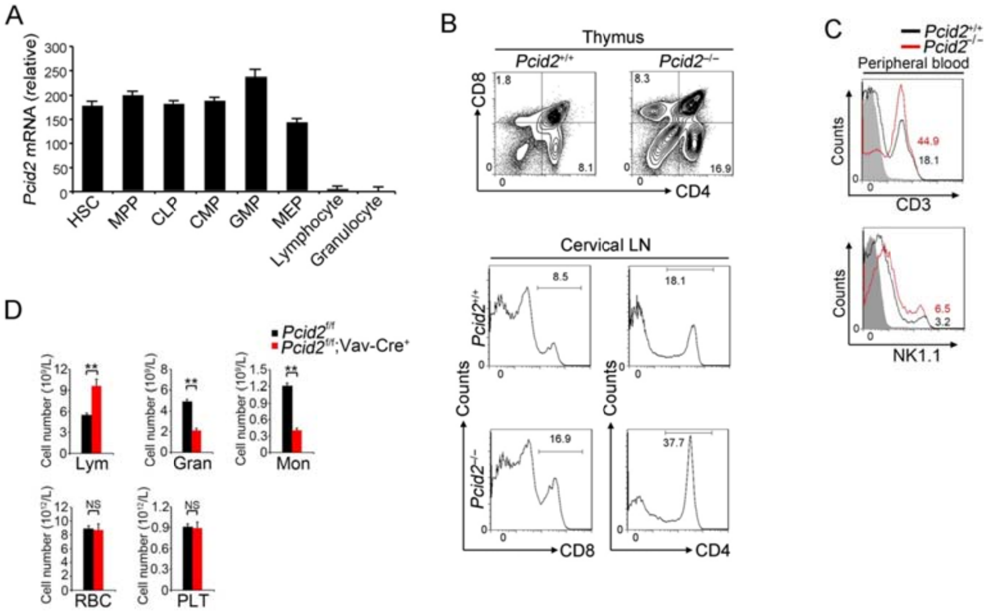

Supplementary Figure 1. *Pcid2* deficiency increases lymphoid cells but decreases myeloid cells. (A) BM hematopoietic progenitors and peripheral blood mature cells were sorted by FACS. Total RNA was extracted from indicated cells and analyzed by real time qPCR. Relative *Pcid2* expression levels were normalized to endogenous *Actb*. Fold changes were compared with *Pcid2* levels in granulocytes. Primer pairs are shown in Supplementary Table S1. (B) Flow cytometry analysis of $CD4^+CD8^-$ T cells and $CD4^-CD8^+$ T cells in thymus and cervical lymph node. LN: lymph node. (C) Flow cytometry analysis of $CD3^+$ T cells and $NK1.1^+$ NK cells in PBMCs. (D) Peripheral blood cell counts were obtained for *Pcid2*$^{f/f}$ and *Pcid2*$^{f/f}$;Vav-Cre mice. Lymphocytes (Lym), granulocytes (Gran), monocytes (Mon), red blood cells (RBC), and platelets were analyzed by XFA6030 automated hemacytometer (Slpoo). PLT: platelet. Results are shown as means ± S.D. n=6. **, *P*<0.01, NS, no significance. Student's *t*-test was used as statistical analysis.

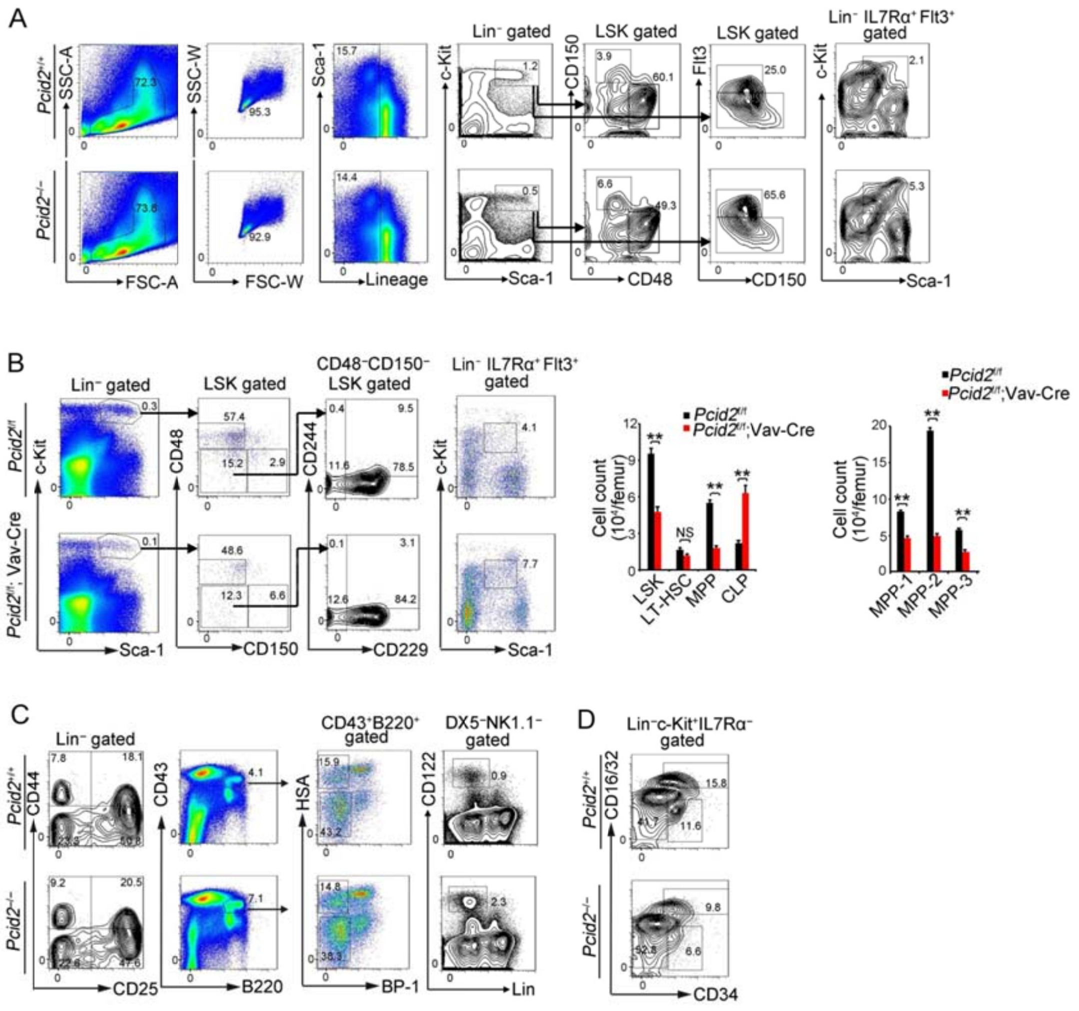

Supplementary Figure 2. *Pcid2*-deficient MPPs prefer to differentiate into lymphoid cells but not myeloid cells. (A) Flow cytometry analysis of LSK (Lin$^-$Sca-1$^+$c-Kit$^+$), LT-HSC (Lin$^-$Sca-1$^+$c-Kit$^+$CD48$^-$CD150$^+$), LMPP (Lin$^-$Sca-1$^+$c-Kit$^+$CD150$^-$Flt3$^+$), MPP (Lin$^-$Sca-1$^+$c-Kit$^+$CD48$^+$CD150$^-$), and CLP (Lin$^-$CD127$^+$Sca-1^{low}c-Kit^{low}) from BM of *Pcid2*$^{f/f}$;Vav-Cre mice and littermate control mice.. (B) Flow cytometry analysis of LSK, MPP, CLP, MPP-1 (CD150$^-$CD48$^-$CD229$^-$CD244$^-$LSK), MPP-2 (CD150$^-$CD48$^-$CD229$^+$CD244$^-$ LSK), and MPP-3 (CD150$^-$CD48$^-$CD229$^+$CD244$^+$LSK) from BM of *Pcid2*$^{f/f}$;Vav-Cre mice and littermate control mice. (C) Flow cytometry analysis of DN1(CD44$^+$CD25$^-$), DN2 (CD44$^+$CD25$^+$), DN3 (CD44$^-$CD25$^+$), DN4 (CD44$^-$CD25$^-$) subsets from mouse thymus, as well as pre-pro B (B220$^+$CD43$^+$BP-1$^-$HSA$^-$), pro B ((B220$^+$CD43$^+$BP-1$^-$HAS$^+$), and NK cell progenitors (NKP, Lin$^-$CD122$^+$DX5$^-$NK1.1$^-$) from BM. (D) Flow cytometry analysis of MEPs (Lin$^-$c-Kit$^+$Sca-1$^-$CD34$^-$CD16/32$^-$) and GMPs (Lin$^-$c-Kit$^+$Sca-1$^-$CD34$^+$CD16/32$^+$) from BM. Student's *t*-test was used as statistical analysis. Data are representative of three independent experiments.

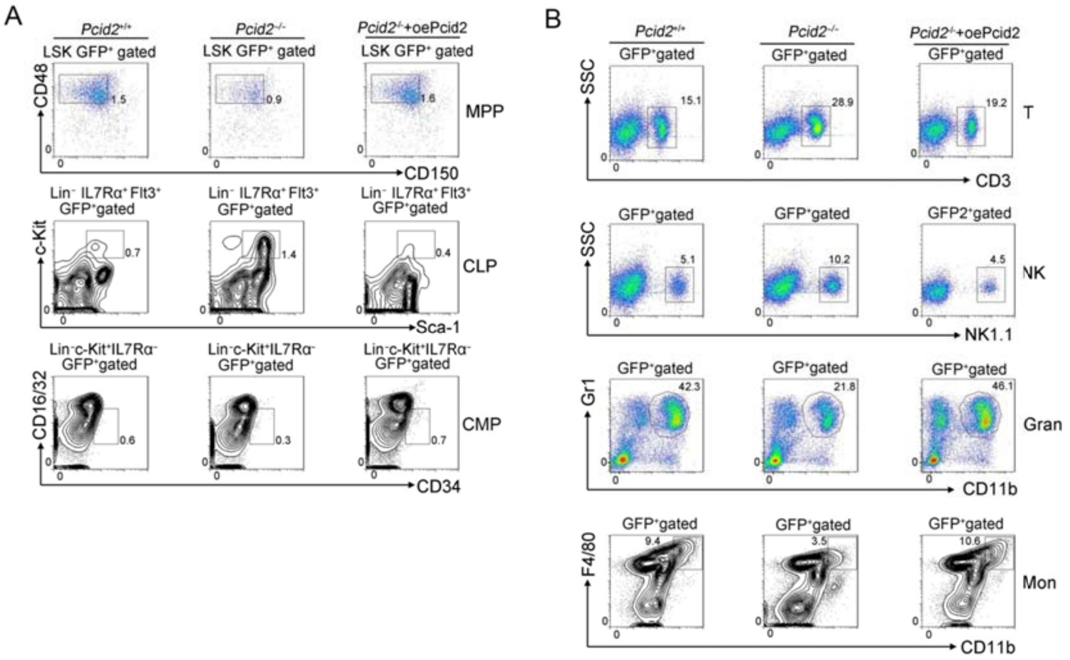

Supplementary Figure 3. *Pcid2* deficiency mediates bias of lymphoid lineage commitment in vivo. (A) $1x10^3$ HSCs were isolated from BM of *Pcid2*$^{+/+}$ or *Pcid2*$^{-/-}$ mice and infected with pMY-GFP-Pcid2 (oePcid2) or empty vector containing retrovirus and transplanted into lethally irradiated recipient mice together with $5x10^6$ helper cells. BM progenitor populations from donor mice (GFP$^+$) were analyzed 8 weeks post transplantation by flow cytometry. (B) Peripheral blood cell populations from donor mice were analyzed 8 weeks post transplantation by flow cytometry. Data are representative of at least three independent experiments.

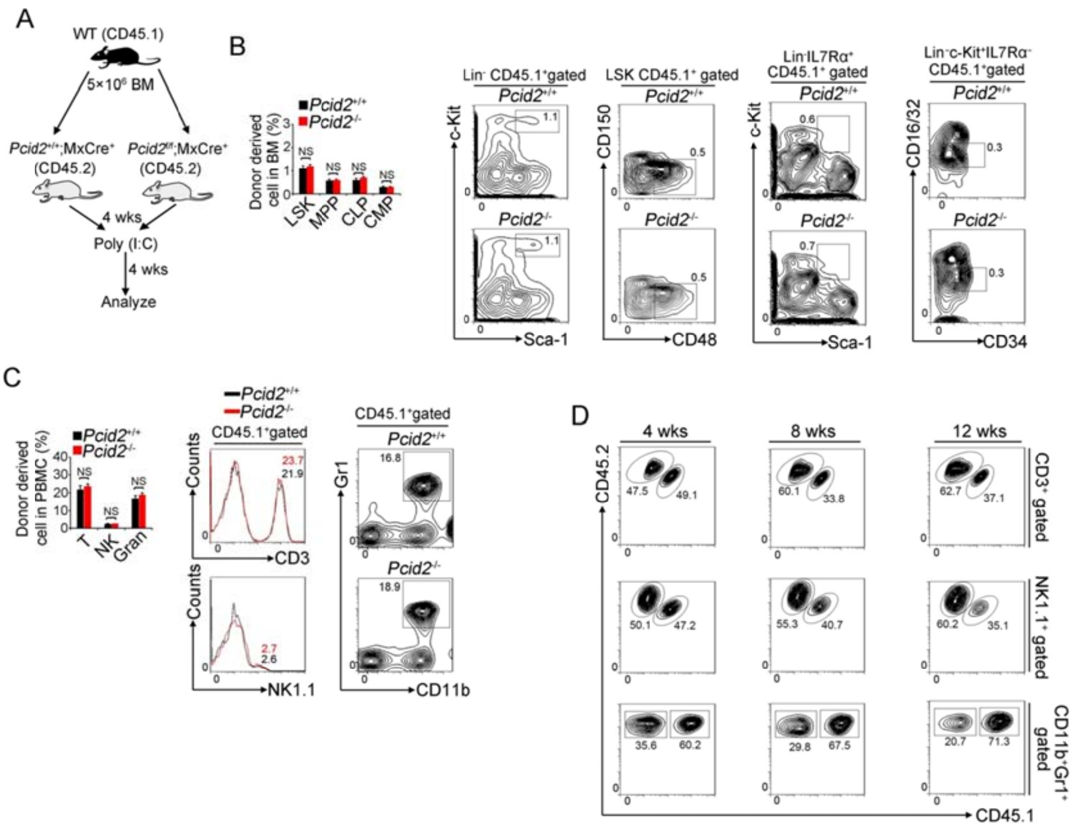

Supplementary Figure 4 *Pcid2* deficiency-mediated bias of lymphoid lineage commitment is cell intrinsic. (A) Schematic representation of reciprocal transplantation. BM cell populations of recipient mice were analyzed 8 weeks post transplantation. (B) Percentages of indicated progenitor cells were analyzed by flow cytometry (left panel) and shown as means ± S.D (right panel). n=6 for each group. NS, no significance. (C) Percentages of indicated peripheral blood cells were analyzed by flow cytometry (left panel) and shown as means ± S.D (right panel). n=6 for each group. (D) 1:1 mixture of $CD45.1^{+}CD45.2^{+}$ wild-type and $CD45.2^{+}$ *Pcid2*$^{-/-}$ HSCs was transplanted into lethally irradiated (10Gy) $CD45.1^{+}/CD45.2^{+}$ recipients together with $5x10^{6}$ helper BM cells for competitive BM transplantation. BM cell populations of recipient mice were analyzed 16 weeks post transplantation by flow cytometry. Student's *t*-test was used as statistical analysis.

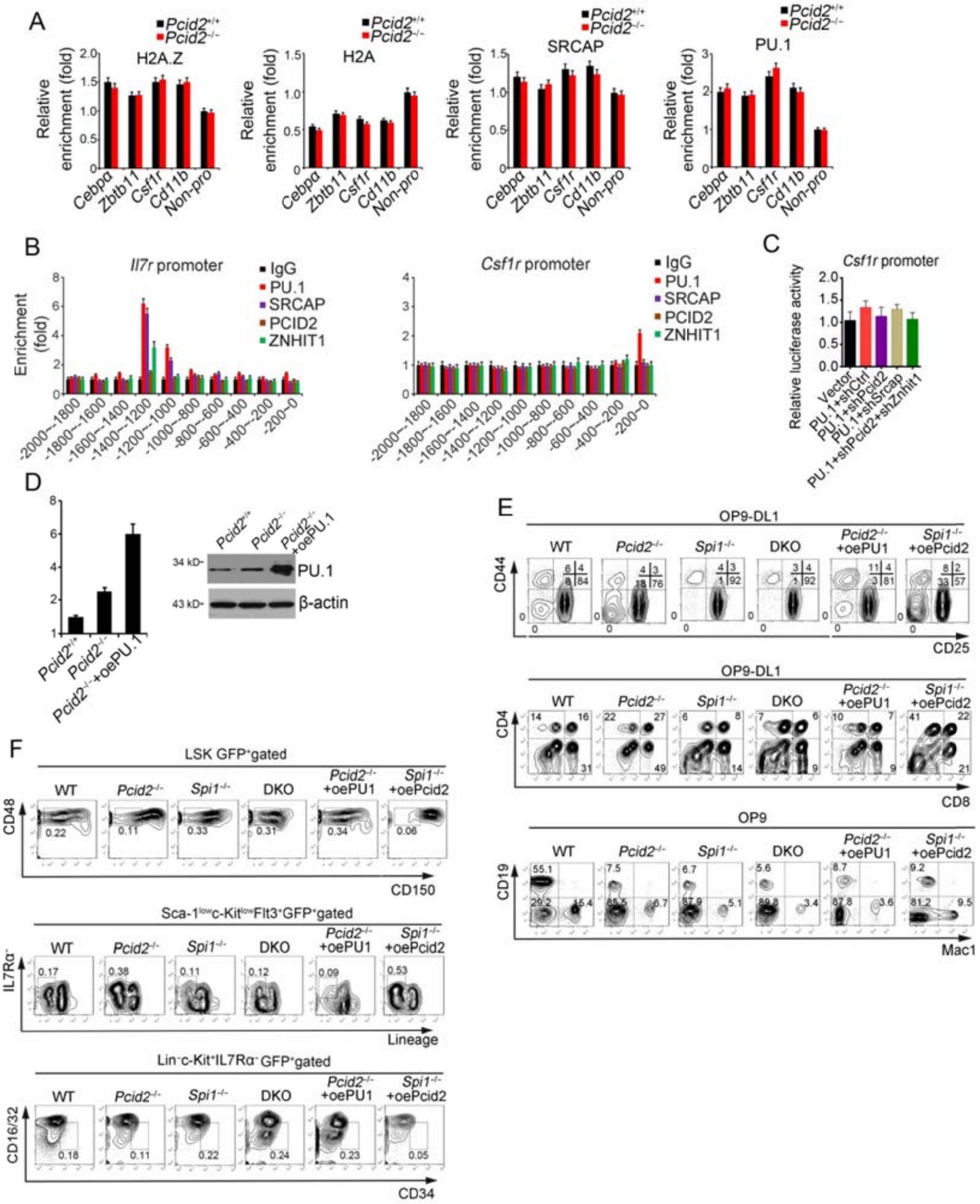

Supplementary Figure 5. ***Pcid2*** **deficiency causes chromatin accessibility of lymphoid determination genes but not myeloid determination gene in MPPs.** (A) Sorted MPPs were lysed for ChIP assays. Indicated myeloid gene promoters were examined by real time qPCR as in Fig. 5A. Non-promoter locus was used as a negative control. (B) Sorted MPPs were lysed for ChIP assays for promoter region mapping. Indicated promoter regions were detected by real time qPCR as in Fig. 5A. IgG was used as a negative control. (C) Flag-PU.1, pTK and pGL3-*Csf1r* promoter together with indicated shRNAs were transfected into 293T cells for luciferase assays. (D) PU.1 was overexpressed in *Pcid2*$^{-/-}$ HSCs, PU.1 expression levels were confirmed by qPCR and immunoblotting. oe, overexpression. (E) In vitro differentiation assays of indicated MPPs were conducted and analyzed by flow cytometry. (F) Indicated HSCs as described in Fig. 5J were sorted, infected and transplanted for 16 wks. Percentages of MPPs, CLPs and CMPs from BM of indicated mice were analyzed by flow cytometry. Data are

representative of at least three independent experiments.

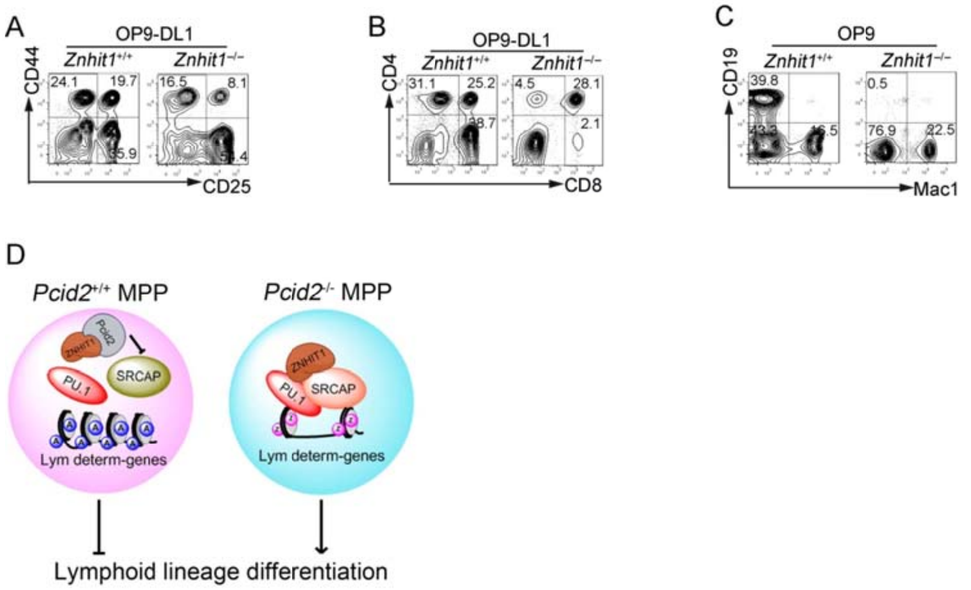

Supplementary Figure 6. *Znhit1* deletion abrogates H2A/H2A.Z exchange to disrupt skewed lymphoid lineage commitment. (A-C) In vitro differentiation assays of indicated LMPPs were conducted and analyzed by flow cytometry. Data are representative of at least three independent experiments. (D) A work model of Pcid2-mediated restriction of lymphoid lineage commitment. In *Pcid2*$^{+/+}$ MPPs, Pcid2 binds to ZNHIT1 to block the SRCAP complex assembly leading to prevention of H2A/H2A.Z exchange at the lymphoid determination genes, which deposits H2A (A) onto the nucleosomes of lymphoid determination genes to suppress the expression of lymphoid lineage genes. In *Pcid2*$^{-/-}$ MPPs, freed ZNHIT1 assembles the SRCAP complex to enrich H2A.Z (Z) onto the lymphoid determination genes, which initiates their expression to drive lymphoid lineage commitment. A, H2A; Z, H2A.Z; Lym determ-genes, lymphoid determination genes.

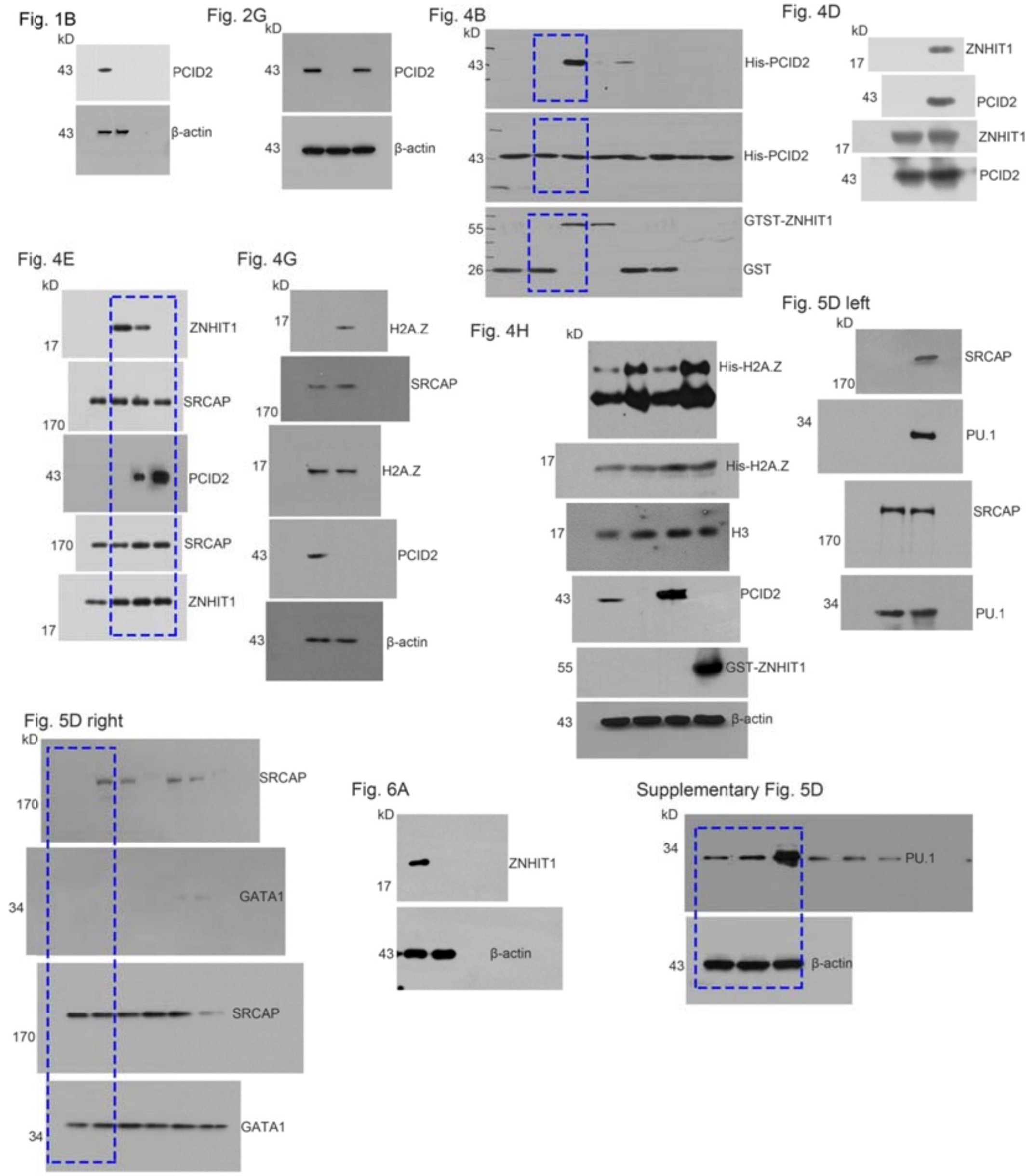

Supplementary Figure 7. Uncropped images of western blotting in this study.

Supplementary table 1. Primer oligonucleotides for qPCR and ChIP-qPCR assays in this study

Genes	Forward	Reverse
mβ-actin	5'- TGACGGGGTCACCCACACTGTGCCCATCTA-3'	5'- CTAGAAGCATTTGCGGTGGACGATGGAGG
mPcid2	5'- ATGGCGCACATCACCATT-3'	5'- GTCGTGATTCCCCACAGC-3'
mZnhit1	5'- CTCGAGTTACACGGTCCACTTCAGACA-3'	5'- AAGCTTATGCAGACGGCGAGACAAGTT-3'
mIl7r	5'- AAGTGGAAATGCCCAGGAT-3'	5'- TTGACTTCCATCCACTTCCA-3'
mIkzf1	5'- GCTGGCTCTCAAGGAGGAG-3'	5'- CGCACTTGTACACCTTCAGC-3'
mSox4	5'- AAGATCATGGAGCAGTCGCC-3'	5'- CCGACTTCACCTTCTTTCGC-3'
mId3	5'- GCATGGATGAGCTTCGATCTTAAC-3'	5'- TTCTCTCGGGCTCCAGGTC-3'
mMaf	5'- AGGATGGCTTCAGAACTGGC -3'	5'- TGCTCATCCATGTAGGTGTGG-3'
mFos	5'- TACTACCATTCCCCAGCCGA-3'	5'- GCTGTCACCGTGGGGATAAA-3'
mSpi1	5'-ATGTTACAGGCGTGCAAAATGG-3'	5'- TGATCGCTATGGCTTTCTCCA-3'
mIl3r	5'- CCCGCTGCTCATTCCAGTC-3'	5'- CACATCACGCCAGAACATCC-3'
mCsf1r	5'- TGACCACAAGAAACGCGACC -3'	5'- ATCAGACAGGGCAGCACAGC -3'
mGata1	5'- TGGGGACCTCAGAACCCTTG-3'	5'- GGCTGCATTTGGGGAAGTG-3'
mZbtb11	5'- ATGACCTGAGCAACTTCACC -3'	5'- CTTGTGCGCTTTGTACTCC -3'
mItgam	5'- CTAAGACAGAGACCAAAGTGG -3'	5'- CAATGCATGGAGAAAAGG -3'
mCebpα	5'- CAAGAACAGCAACGAGTACCG-3'	5'- GTCACTGGTCAACTCCAGCAC-3'
mIl7r pro	5'- GCAGTTAAGTTCAGGAGCTTCAGG-3'	5'- GAAGCACGGTTGTATGTGCAAGTG-3'
mIkzf1 pro	5'- GCGAAAGCCTGGTCTGAG-3'	5'- CGCGCAGTCACTTGTTAGAG-3'
mId3 pro	5'- GGTCCATGCTTTTTCTTTCTCCGTGGAAAAGG-3'	5'- GGGAAAAAATTAATTGCGGTGAAGCTGAG[illegible]
mSox4 pro	5'- CGGGAGACAATGGGTAAGAA-3'	5'- CCAAAGGATAGATGGGTTCG-3'
mMaf pro	5'- TTTCTATACTATTATGCTAATCGCTGCCGC-3'	5'- CGAGAAGAGTTTAAAGCAATTGCTGAGTTT
mFos pro	5'- CCCCCTAAGATCCCAAATGT-3'	5'- GTCGCGGTTGGAGTAGTAGG-3'
mCebpα pro	5'-GCCTAACCACGGACCACGTGTGT -GCGGGGGCGACAGCG-3'	5'- TGACTTTCCAAGGCGGTGAGTGGG-3'
mZbtb11 pro	5'- GACAGCAACCCCTTTAACG-3'	5'- GCTGAGAACCATAGCTCTAACC-3'
mItgam pro	5'- GGACCTTCTCTTCAGGTCTCC-3'	5'- ATTGAAAGCACCCCTATTCG-3'
mCsf1r pro	5'- GGGCAGATGAGAAAGGTATGA-3'	5'- AGTCTCCCAGATGAGCAGTGA-3'
Non pro	5'- CGCGTTTCTTTGATCAATCC -3'	5'- GAGCAAGCACCCTTAAACCA -3'
mIl7r -2000	5'- AAGACAACCCATTTTGGATACC-3'	5'- ATTATCCCCCAATGCTGC-3'
mIl7r -1800	5'- TCGAAAAATCCAGACTGACC-3'	5'- GACATTATCCCCCAATGC-3'
mIl7r -1600	5'- GCTCAAGTATTCCCCTGC-3'	5'- TGTGTTAATGGTGTCAGTCG-3'
mIl7r -1400	5'- CCCTGACCAAAGGATTACC-3'	5'- ACAGACCAACCAAAGAAGC-3'
mIl7r -1200	5'- CACTTACAGTACTAGAACATGG-3'	5'- GAAGCACACTCACTCTAGC-3'
mIl7r -1000	5'- TGCATGGCTAAGCACTGC-3'	5'- AAGGACACTTGAACACTCAACC-3'
mIl7r -800	5'- TGCACACTGGGGATTTGACC-3'	5'- TGTCAGCGCCATCTTGTAACG-3'
mIl7r -600	5'- AATTCTCCTTAAAAGGGACG-3'	5'- GAGCAAGAGAGCAAGAGC-3'
mIl7r -400	5'- GCTCTCTTGCGCTCTGGCTC-3'	5'- CTTACACGCGTTCACGAC-3'
mIl7r -200	5'- CCAGTGTGTTGGGATATCC-3'	5'- GACAGCAAGACCATCTAACAGCAG-3'
mCsf1r-2000	5'- TGTAGTAACTGCCCCACC-3'	5'- TTTCCTCATTCTCAGAAGC-3'

mCsf1r-1800	5’- TCATGTTAGGTCAGAGTGC-3’	5’- CACCATACTTTCAGCTCC-3’
mCsf1r-1600	5’- GCTAAGACAGAGGCAGGAGC-3’	5’- ATGAAAAAGACGAGTATCGAGG-3’
mCsf1r-1400	5’- ACTCTCATCCCCAAGTGG-3’	5’- TGTATGGTAGCACGCATAGC-3’
mCsf1r-1200	5’- AGCTGACAAGTCACCACG-3’	5’- GCCTTCATCTCTCTGAAACC-3’
mCsf1r-1000	5’- ATCTCAGCCCTCGATACTCG-3’	5’- TCCAGTCAGCTCTGTCATCC-3’
mCsf1r-800	5’- TCTTATCAAACTCCCAGG-3’	5’- CCACCAGTAGAAAGATCC-3’
mCsf1r-600	5’- CACTGAGAACCCATTACC-3’	5’- GGAGGAAGTGAGAGTAAGG-3’
mCsf1r-400	5’- CCTGAGAGCCTAGCTGGGTCC-3’	5’- GGAATGGAATCTGAGCTGGCC-3’
mCsf1r-200	5’- GGTTTCTACTCATCCCGTCATCA-3’	5’- GGATGGAGAGGTAGATTC-3’

M, mouse.
